# INTENTIONAL EVALUATION OF DYADIC BENEFITS OF EARLY-PHASE INTERVENTIONS: ROLES OF PRETEST RISK AND RELATIONAL IMPACT

**DOI:** 10.1093/geroni/igae098.1867

**Published:** 2024-12-31

**Authors:** Karen Lyons, Luke Russell, Kalisha Bonds Johnson, Glenna Brewster, Lyndsey Miller

**Affiliations:** Boston College, Chestnut Hill, Massachusetts, United States; Illinois State University, Normal, Illinois, United States; Emory University, Atlanta, Georgia, United States; Emory University, Atlanta, Georgia, United States; Oregon Health & Science University, Portland, Oregon, United States

## Abstract

The evaluation of early-phase (i.e., Stage I, NIH Stage Model), small sample dyadic interventions is still primarily focused on testing separate effects for each member of the older adult-care partner dyad, rather than the benefits for dyads as a whole. Moreover, there is little attention to assessing pre-test risk status within dyads (especially in quasi-experimental or single-arm designs) or the relational impact of interventions. We first examined pre-test risk status across several outcomes (e.g., depressive symptoms, self-efficacy, communication) in 39 dyads and found 37-68% of older adults with chronic illness were at-risk at baseline; 40-79% of care partners; and 15-53% of dyads. Second, we examined dyadic benefits of a health behavior intervention, using two approaches, and found above zero approaches may deflate dyadic benefits compared to pre-test risk approaches. We used a pattern analysis to examine benefits across outcomes and within dyads and found 40% of dyads who received the intervention had expected positive outcomes for depressive symptoms and self-efficacy; 20% had expected positive scores for depressive symptoms, self-efficacy and aerobic exercise; and 13% had expected positive scores for all five target outcomes. Dyads where both members had highest pre-test risk level did not experience expected dyadic benefits. Finally, although we did not find strong evidence the intervention improved communication compared to a wait-list condition, there was no evidence it resulted in worse communication. Implications of using each approach and selecting different benchmarks for defining success are discussed. Results provide rationale for more intentional evaluation of small-sample, early-phase dyadic interventions.

